# Correction to: Assessing the utility and efficacy of e-OSCE among undergraduate medical students during the COVID-19 pandemic

**DOI:** 10.1186/s12909-022-03285-y

**Published:** 2022-04-01

**Authors:** Sarra Shorbagi, Nabil Sulaiman, Ahmad Hasswan, Mujtaba Kaouas, Mona M. Al-Dijani, Rania Adil El-hussein, Mada Talal Daghistani, Shumoos Nugud, Salman Yousuf Guraya

**Affiliations:** 1 Department of Family and Community Medicine and Behavioural Science, College of Medicine, University of Sharjah, Sharjah, United Arab Emirates; 2 Baker/ IDI Heart and Diabetes Institute, 75 Commercial Road, Melbourne, VIC 3004 Australia; 3 Clinical Sciences Department, College of Medicine, University of Sharjah, Sharjah, United Arab Emirates

**Correction to: BMC Med Educ 22, 156 (2022)**

**https://doi.org/10.1186/s12909-022-03218-9**

Following publication of the original article [1], the have been notified by the authors that some corrections were missed.

1. The capitation of Fig. 2 has been corrected.

2. A few other minor corrections in the body of the text.

The original article has been corrected.
